# Managing Japanese Encephalitis Virus as a Veterinary Infectious Disease Through Animal Surveillance and One Health Control Strategies

**DOI:** 10.3390/life15081260

**Published:** 2025-08-07

**Authors:** Jae-Yeon Park, Hye-Mi Lee

**Affiliations:** College of Veterinary Medicine, Chungnam National University, Daejeon 34134, Republic of Korea; wodus5818@naver.com

**Keywords:** Japanese encephalitis virus, veterinary infectious disease, zoonotic transmission, animal surveillance, One Health

## Abstract

Japanese encephalitis virus (JEV) is a mosquito-borne zoonotic flavivirus that circulates primarily within animal populations and occasionally spills over to humans, causing severe neurological disease. While humans are terminal hosts, veterinary species such as pigs and birds play essential roles in viral amplification and maintenance, making JEV fundamentally a veterinary infectious disease with zoonotic potential. This review summarizes the current understanding of JEV transmission dynamics from a veterinary and ecological perspective, emphasizing the roles of amplifying hosts and animal surveillance in controlling viral circulation. Recent genotype shifts and viral evolution have raised concerns regarding vaccine effectiveness and regional emergence. National surveillance systems and animal-based monitoring strategies are examined for their predictive value in detecting outbreaks early. Veterinary and human vaccination strategies are also reviewed, highlighting the importance of integrated One Health approaches. Advances in modeling and climate-responsive surveillance further underscore the dynamic and evolving landscape of JEV transmission. By managing the infection in animal reservoirs, veterinary interventions form the foundation of sustainable zoonotic disease control.

## 1. Introduction

Japanese encephalitis virus (JEV) is a mosquito-borne zoonotic flavivirus that causes Japanese encephalitis (JE), a potentially severe neuroinvasive disease in humans [1,2]. The virus is endemic in many parts of Asia and the Western Pacific, where it remains a significant public health threat, with an estimated 100,000 clinical cases reported annually [3]. Most cases occur in children and adolescents living in rural areas [3]. Although human vaccination programs have reduced disease incidence in some countries, sporadic outbreaks and sustained enzootic transmission continue [4].

JEV maintains its transmission cycle primarily through complex interactions among vectors, amplifying hosts, and incidental hosts. *Culex* (*Cx.*) species mosquitoes, particularly *Cx. Tritaeniorhynchus*, are the primary vectors of these diseases [5,6]. Among vertebrate hosts, pigs are major amplifying hosts because of their high viremia and proximity to human habitats [7,8]. Ardeid birds, such as herons and egrets, are important reservoirs and contribute to long-distance viral dissemination [5]. Humans are considered dead-end hosts because they do not develop sufficient viremia to infect feeding mosquito populations [5]. Thus, JEV is fundamentally a veterinary infectious disease maintained in animal populations, with humans affected through zoonotic spillover.

In addition to the complex ecology of JEV, the virus exhibits considerable genetic diversity and is classified into five genotypes (G) I–V based on envelope gene sequences [9,10]. Historically, GIII was predominant in endemic regions until the late 20th century [11]; however, GI has progressively replaced GIII in East and Southeast Asia [12]. More recently, GV strains have been detected in China and South Korea [7,13,14], and GIV caused the first large outbreak outside Southeast Asia in northern and eastern Australia during 2021–2022 [15,16]. These emerging genotypes raise concerns about viral fitness, geographical expansion, and potential impacts on vaccine-induced immunity, underscoring the need for genomic surveillance for JEV control [17,18].

The veterinary–human interface is critical for zoonotic spillover events. Managing infection in animal reservoirs, particularly pigs and birds, is essential for breaking the transmission cycle before the virus reaches human populations. Agricultural intensification, land use changes, and peri-urban expansion have increased the contact between wildlife, domestic animals, and humans, elevating the risk of zoonotic transmission [19,20]. Recent global mapping and modeling efforts underscore the need to understand JEV transmission dynamics in broader ecological and veterinary contexts.

This review summarizes the current understanding of JEV transmission from a veterinary infectious disease perspective, highlighting the roles of amplifying and reservoir hosts in sustaining viral circulation. It also explores how animal-focused strategies, including surveillance and vaccination, contribute to the reduction in human disease risk. We further emphasize the importance of integrated surveillance and control efforts within a One Health framework, where veterinary, environmental, and public health sectors collaboratively address the challenge of zoonotic vector-borne diseases such as JE.

## 2. Veterinary Hosts and Their Role in Transmission

### 2.1. Pigs as Amplifiers and Birds as Reservoirs

The primary vertebrate hosts in the JEV transmission cycle are domestic pigs and ardeid birds, which have fully distinct ecological roles [21]. Pigs are recognized as the principal amplifying hosts, owing to their ability to develop high-titer viremia after infection, which is sufficient to infect feeding mosquitoes. After a brief incubation period of 1–3 days, pigs exhibit viremia that can persist for 3–5 days [22], during which they can infect large numbers of mosquitoes without showing overt clinical symptoms [21,23]. The natural infection rate in pigs can reach 98–100%. In piglets, viremia can be detected within 24 h of infection, lasting up to 6 days [22,24]. Although most infections in pigs are subclinical, reproductive disorders are well documented in breeding sows [25]. These manifestations not only affect livestock productivity but also serve as indirect indicators of JEV circulation in endemic regions [19]. The common practice of rearing pigs close to human habitation in many parts of Asia facilitates zoonotic spillover, particularly in the context of poor biosecurity [19].

**Figure 1:**
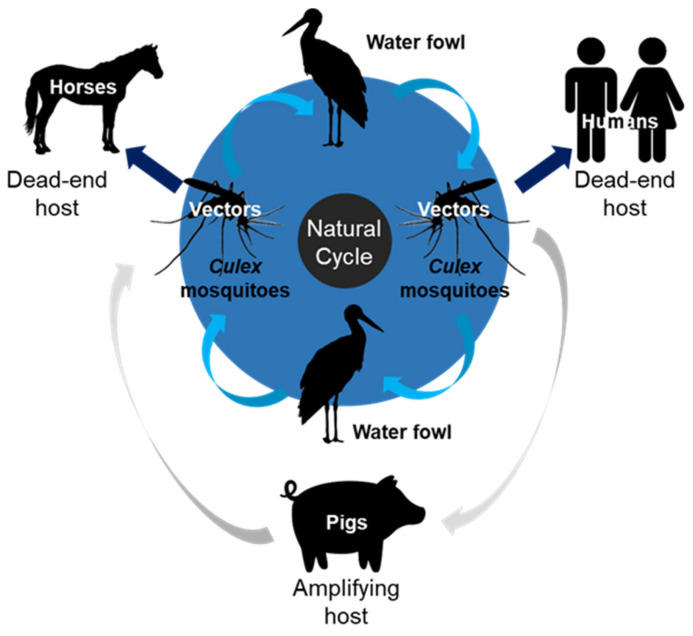
Schematic diagram of JEV transmission cycle.

Ardeid birds, including herons and egrets, act as natural reservoir hosts for these parasites [26]. They play a central role in maintaining endemic transmission through asymptomatic infections and sustained viremia, which supports continuous virus–mosquito–bird cycles independent of pig populations [19,27]. Notably, many ardeid species are migratory, enabling the potential long-distance dissemination of the virus across national and continental borders [19]. This underscores their significance not only in local maintenance but also in the geographical spread of JEV to previously unaffected regions [19,28]. Birds may also play a crucial role in the genotypic shift of JEV, as the newly emerged JEV GI replicates more efficiently than JEV GIII in avian-derived cells and ducklings/chickens [11,27]. The ecological interface among pigs, birds, and mosquitoes forms the foundation of JEV transmission [3]. Monitoring the dynamics of these animal populations is essential for anticipating outbreaks in humans (Figure 1).

### 2.2. Infection Mechanism and Clinical Presentation in Hosts

In both pigs and birds, JEV infection generally follows a short viremic phase during which the virus replicates in endothelial cells, monocytes, and lymphoid tissues [8,23]. The virus then disseminates systemically, although it rarely causes neurological symptoms in these animals [7]. In pigs, the most clinically relevant outcomes are related to reproduction, as mentioned above. However, post-weaning piglets may also exhibit transient fever and lethargy, albeit infrequently [8,23]. Infected suckling piglets can develop encephalitis, exhibiting tremors and convulsions, and the mortality rate in naïve infected suckling piglets can be almost 100% [8].

In birds, experimental infections have demonstrated that ardeid species can support viremia levels sufficient to infect *Cx.* mosquitoes without showing disease, indicating effective viral tolerance [29]. This differs from other avian species, such as chickens, which develop lower viremia and are thus considered poor reservoirs of the virus [19,26,30]. The virus can be detected in various organs of infected birds, including the brain, heart, liver, spleen, and kidneys [27].

The lack of overt symptoms in most veterinary hosts contributes to the silent spread of JEV in endemic areas [8]. Therefore, surveillance systems that rely solely on clinical presentations are inadequate. Instead, serological surveys of pig populations, particularly piglets, have been employed as early indicators of active viral circulation [9,10,31]. JEV has also been isolated from rodents and bats, but their precise roles as maintenance or amplifying hosts remain under investigation [27].

### 2.3. Transmission Pathway from Animals to Humans

JEV transmission to humans occurs indirectly through a bridge vector system, most commonly involving *Cx. tritaeniorhynchus*, a mosquito species that thrives in rice paddies and can feed on both animals and humans [7,21]. After feeding on a viremic pig or bird, mosquitoes undergo an extrinsic incubation period of approximately 10–14 days, after which they can transmit the virus to susceptible hosts [32]. In Australia, *Cx. annulirostris* is considered the principal JEV vector [19,33]. Mosquito JEV infection rates tend to be higher near pig farms [34].

Humans, as dead-end hosts, do not develop viremia at levels sufficient to sustain further transmissions [19,28]. However, infections can lead to severe clinical outcomes, including encephalitis, paralysis, and death. Globally, approximately 68,000–70,000 cases of JE and 10,000–20,000 deaths occur annually [1]. Approximately 20–30% of patients die, and 30–50% of survivors experience severe neuropsychiatric sequelae [1]. The risk of human infection is directly correlated with the density of infected mosquitoes and the presence of amplifying hosts in the surrounding environment [19]. Outbreaks typically occur in rural and peri-urban areas during the rainy season, when vector populations surge [35].

Although mosquito-borne transmission is the primary route, JEV transmission between pigs without a vector has also been experimentally confirmed [7,23]. Infected pigs can shed the virus via oral–nasal routes, which may contribute to virus maintenance within pig herds [23]. In humans, rare cases of JEV transmission through contaminated blood and solid-organ donations have been reported [19]. Land use changes, agricultural intensification, and urban expansion have increased the frequency and intensity of contact between animal reservoirs and human populations [36]. These anthropogenic factors have reshaped traditional transmission landscapes, making it increasingly important to consider veterinary hosts not only in animal health contexts but also in human public health planning [21].

Integrated vector management, pig vaccination in endemic zones, and environmental monitoring of bird populations have all been proposed as components of a One Health–oriented JEV control strategy [19,37,38]. Importantly, reducing viral circulation in animal hosts can reduce the force of infection in mosquito vectors, thereby sustainably lowering the risk to humans [9,39].

## 3. Surveillance Systems and Public Health Implications

### 3.1. National Animal Surveillance Strategies

National animal surveillance strategies for JEV are critical for monitoring viral activity, especially in pigs and other sentinel animals [8,40,41]. These systems function as essential elements of early warning networks and vector control programs (Table 1) [42,43].

Several Asian countries where JEV is endemic have established animal-based surveillance systems [8,42,43]. For instance, Japan has had a robust sentinel surveillance system since 1965 [8,44]. This system involves monitoring seroconversion rates in sentinel pigs, which often record high seroconversion rates annually, suggesting the presence of JEV-infected mosquitoes during summer in most parts of Japan [53]. These data help authorities anticipate outbreak seasons. Detection of seroconversion in pig herds, especially during the early summer months, provides timely indicators of JEV circulation and precedes human case reports by several weeks [54,55,56]. Japan also monitors vector populations and conducts active virus isolation in mosquitoes, forming an integrated animal-vector surveillance model.

In Vietnam, JEV is recognized as a significant public health concern, and although a human immunization program was initiated in 1997, accurate surveillance data are crucial for evaluating and guiding the program [57]. In 2016, 92% of the 24 countries with JEV transmission risk conducted JEV surveillance, an increase from 75% in 2012 [42]. Among them, 58% conducted national JEV surveillance and 46% conducted sentinel surveillance [42]. Countries with sentinel surveillance had a median of eight sites (range, 1–223) [42]. Most countries with JEV surveillance have confirmed suspected cases using JEV-specific diagnostic testing of serum and cerebrospinal fluid [42]. Indonesia has also confirmed JEV as an endemic human disease through sentinel surveillance, providing a basis for ongoing disease burden information and monitoring the impact of vaccine introduction [58].

South Korea employs a similar approach, whereby blood samples from pigs, particularly those aged 2–6 months, are collected regularly for JEV antibody testing. These data are combined with vector monitoring data from mosquito traps to assess regional transmission risk [8]. Moreover, South Korea has explored the inclusion of sentinel chickens in JEV surveillance, although their lower viremia has limited utility compared to that of pigs [8].

In China, surveillance is integrated into broader arboviral monitoring. Serological testing in pigs is conducted seasonally in endemic provinces, and virus detection in mosquito pools is routinely performed. Some regions have initiated environmental surveillance of migratory birds, which act as reservoirs and potential long-distance disseminators of viruses [59]. Collectively, these strategies illustrate the diversity and evolution of animal surveillance systems in JEV-endemic areas. Pigs remain the cornerstone species for monitoring because of their high viremia helplessness and close contact with humans.

### 3.2. Predictive Value for Human Risk

Animal surveillance has valuable predictive capabilities for human JEV outbreaks. Studies have shown that an increase in JEV antibody detection rates among pigs coincides with the highest rate of virus isolation from mosquitoes [48,60]. The outbreak among pigs can precede human outbreaks by approximately three weeks [61]. For example, in suburban Bangkok, human cases occurred approximately two months after the first JEV isolate was found in mosquitoes and one month after mass JEV seroconversion in pigs [44]. For instance, in Japan, annual pig surveillance has allowed authorities to accurately anticipate outbreak seasons. Local human vaccination schedules are often adjusted based on early seropositivity data from pigs [44,54]. Similarly, in South Korea, seroprevalence rates in pig farms have shown a strong correlation with regional human case burdens, providing quantitative data to guide vector control priorities [62].

In India, monitoring mosquito abundance and pig JEV seropositivity can help predict JEV outbreaks in the human population [6,63]. A study in Assam found a good correlation between mosquito numbers and JEV positivity in pigs and humans and between pig and human cases (*p* < 0.05), with the highest activity during the monsoon season [63]. Another study in Mandya district, India, used domestic pigs as sentinels and found seroconversion to JEV in 44 pigs across five localities, indicating a pig–mosquito cycle operating throughout the year [64,65]. In Saipan, a high seroprevalence of 96% was found in pigs during a 1990 encephalitis outbreak, suggesting the recent introduction of the virus [66,67]. However, subsequent surveillance in 1991 showed no evidence of ongoing JEV transmission [66].

The use of insecticide-treated mosquito nets has also been shown to significantly reduce JEV seroconversion rates in both humans and pigs, demonstrating an effective defense against JEV circulation in endemic areas [8,9,68,69]. However, the predictive power of these systems depends on surveillance frequency, spatial resolution, and coverage [70]. In areas where pig density is low or where sentinel monitoring is irregular, the correlation between animal and human data may diminish.

### 3.3. Limitations and Innovations

Despite their utility, current animal surveillance systems for JEV face limitations, including disadvantages that can compromise their efficacy [71]. One significant challenge is the intensive animal husbandry required [71]. Consequently, attention has been redirected towards vector surveillance [71].

Early detection of emerging foreign animal diseases, such as JEV, is crucial for pathogen surveillance and control programs [72]. There is a recognized need to enhance the quality of JEV surveillance [42]. Although sentinel surveillance has limitations, it provides estimates that enable monitoring of JEV incidence over time [73]. However, a possible disadvantage is that if sentinel hospitals are limited in scope, it can result in incomplete case ascertainment [42,73]. Data required to improve suspected case classification and guide program expansion are often insufficient [42].

Innovations are being explored to overcome these limitations of the current study. The use of mosquito cell cultures has shown a clear advantage over mouse inoculation for isolating JEV [74]. Furthermore, increased pig farming and travel between JEV-endemic areas and previously non-epidemic regions, such as high-altitude Tibet, have highlighted the need for enhanced disease prevention and control strategies, alongside surveillance [75]. In Tibet, JEV infection was found to be prevalent in a high-altitude region that was previously considered to be JEV-free [75]. This underscores the importance of a One Health approach, focusing on the phylogeography of JEV, the distribution and abundance of mosquito vectors, and seroprevalence in humans and animal reservoirs to understand JEV epidemiology in highland areas [37]. Novel mosquito-based surveillance systems also offer a means to investigate the status of JEV in regions where active surveillance has been discontinued [76]. By refining animal-based surveillance systems and better linking them with human health responses, it is possible to develop more proactive rather than reactive JEV control strategies.

## 4. Vaccination Strategies in Animals and Humans

### 4.1. Vaccine Types and Implementation

The prevention of JE relies heavily on vaccination strategies for both humans and animals. Although vaccines are available for both, their implementation differs between human and animal populations (Table 2). Several types of JE vaccines have been developed and licensed for human use [35]. These include mouse-brain-derived killed–inactivated vaccines, cell-culture-derived live–attenuated vaccines, cell-culture-derived killed–inactivated vaccines, and genetically engineered live–attenuated chimeric vaccines [35,77,78,79]. The live–attenuated SA 14-14-2 vaccine is considered safe, effective, and inexpensive [80]. The first licensed JE vaccine was an inactivated mouse-brain-derived vaccine based on the Nakayama JEV strain [77,81,82]. Another mouse-brain-derived vaccine was produced in Japan using the Beijing-1 strain for domestic use [83]. The production of the mouse-brain-derived vaccine (JE-VAX) ceased in 2006, and all remaining stocks expired in 2011 [84].

A live–attenuated cell-culture-derived JE vaccine, SA14-14-2, was developed in China based on an attenuated form of the virulent JEV strain SA 14 [97]. This vaccine was first licensed in China in 1988 and is currently produced in primary hamster kidney (PHK) cells [97]. Over 300 million doses of SA14-14-2 have been administered to Chinese children, demonstrating its excellent safety and efficacy [98]. SA14-14-2 is now the most widely used JE vaccine in endemic areas and has been licensed in other Asian countries, including Japan, China, Taiwan, South Korea, and Thailand [99]. In China, SA14-14-2 is administered to children (9–12 months) in two doses, one year apart, with a booster dose at school-entry age [100]. This vaccine is highly immunogenic, with 85–100% seroconversion rates after a single dose and nearly complete seroconversion rates after two doses administered 1–3 months apart [100]. Case–control studies have indicated high efficacy, with 80–99% protection after one dose and over 98% after two doses [100]. SA14-14-2 appears to be effective and safe when administered in a two-dose regimen, with no severe vaccine-induced adverse events observed [101,102]. In contrast, Japan and South Korea primarily rely on inactivated Vero cell-based vaccines, especially for adults and travelers [62].

A PHK cell-derived inactivated JE vaccine was developed using the Beijing-3 (P3) strain, which has been widely used in China since 1968 and adapted for production in African green monkey kidney (Vero) cells [84,103]. In Japan, another Vero cell-derived inactivated vaccine, produced using the Beijing-1 strain, is available under the trade names JEBIK V and ENCEVAC [104,105]. A new Vero cell-derived, inactivated JE vaccine, IC51, was developed using the attenuated SA 14-14-2 strain and has been licensed in many countries since 2009, including the US, Europe, Canada, Australia, Hong Kong, Switzerland, and India [106,107]. IC51 is formulated with aluminum hydroxide as an adjuvant [106]. Clinical trials have shown that IC51 induces immunogenicity equivalent to or higher than that of JE-VAX, with a more favorable safety and tolerability profile [108]. The pediatric use of IC51 was approved in early 2013 for children aged 2 months to under 16 years in the US and Europe [109]. After a two-dose primary immunization with IC51, seroprotection rates decrease over time, but a booster at 1 or 2 years leads to complete seroconversion [108].

A genetically engineered live–attenuated chimeric JE vaccine, ChimeriVax-JE, was produced using a yellow fever 17D virus backbone [110]. This vaccine is commercially available in Australia and Thailand, where a single dose is recommended for individuals aged ≥ 12 months. Studies have shown that ChimeriVax-JE is safe, immunogenic, and protective in both animal and human models [111,112]. A single dose of IMOJEV generated nearly complete seroconversion (~99%) in adults, similar to three doses of JE-VAX (~95%), with approximately 94% seroconverting within 14 days [112]. The ChimeriVax-JE virus is less likely to be transmitted by mosquitoes from vaccinated individuals to other hosts.

Vaccination in veterinary practice mainly targets domestic pigs to reduce virus amplification and consequent human transmission [113]. Although inactivated vaccines for pigs are available and used in Japan, their widespread adoption is limited by economic and logistical factors [114]. Pig vaccination prevents reproductive losses and lowers viral circulation, thereby reducing transmission risk to humans [1]. Vaccinating pigs leads to antibody production within one week, which is retained for at least 36 days, and no virus has been isolated from vaccinated pigs, unlike in unvaccinated controls.

### 4.2. Indirect Protection Through Pig Vaccination

Vaccinating pigs indirectly protects the human population by decreasing viral amplification and mosquito infection rates [26]. This transmission-blocking effect has been demonstrated in several field studies and modeling simulations [115]. In Japan, mass vaccination of horses has led to a decline in the local prevalence of this disease [116]. Studies in India have shown that sentinel pig seroconversions were significantly associated with human cases four weeks before their occurrence [115]. In Bangladesh, a model suggested that vaccinating 50% of pigs annually could lead to an estimated 82% reduction in annual JEV incidence in pigs [117]. The widespread distribution of historical JEV infections in pigs suggests an important role in virus transmission [7,8]. Studies have also highlighted that keeping pigs in urban areas increases the number of mosquitoes capable of being JEV vectors.

### 4.3. Duration of Immunity and Cost-Effectiveness

The duration of immunity provided by JE vaccines varies depending on the vaccine type and species. In human populations, the live–attenuated SA14-14-2 vaccine offers long-lasting immunity, often protecting for several years after a single dose. Inactivated vaccines generally require multiple doses and periodic boosters to maintain protective antibody levels. Despite these differences, both vaccine types have significantly reduced JE incidence when incorporated into national immunization programs in endemic regions [106,112,118,119,120].

In pigs, the duration of vaccination is relatively short, often requiring seasonal or pre-reproductive immunization schedules [11]. However, routine vaccination of pigs is generally cost-justified only in regions with high pig density and frequent outbreaks [117]. In low-resource agricultural settings, such as Nepal, economic limitations often hinder widespread pig vaccination, even when human immunization programs are active [121]. In contrast, human JE vaccination is highly cost-effective in endemic areas, particularly when it is incorporated into national immunization programs [122,123]. For example, a cost-effectiveness study conducted in Shanghai, China, demonstrated that both inactivated (P3) and live–attenuated (SA14-14-2) JE vaccine strategies were cost-saving compared with no immunization [124]. The SA14-14-2 vaccine strategy resulted in 47% greater savings than the P3 strategy per 100,000 individuals. Integrating animal vaccination into broader JE control programs, alongside vector control and human immunization, offers a comprehensive and potentially cost-effective strategy within a One Health framework. Cost-effectiveness analyses suggest that while human vaccination has immediate public health benefits, veterinary vaccination contributes to long-term ecological control by disrupting transmission at its animal source.

## 5. Integrated One Health Control Approaches

### 5.1. Cross-Sector Integration Examples

**Figure 2:**
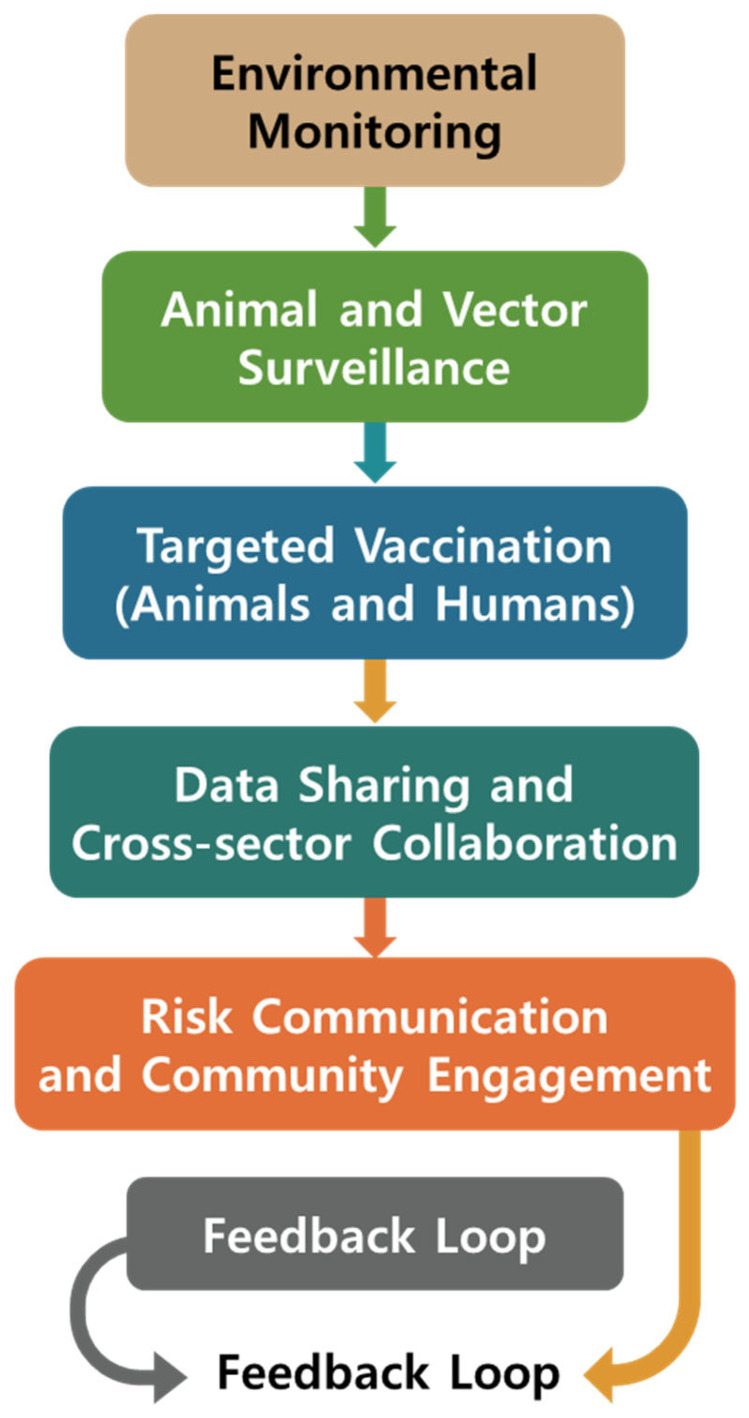
One Health-based integrated management flow of JE.

Integrated control of JE requires coordinated efforts across veterinary, environmental, and human health sectors. Veterinary surveillance data, notably pig serosurveillance, have enhanced early viral detection and informed public health interventions in endemic countries such as Japan [31]. These data are systematically shared with local health departments to inform mosquito control campaigns and guide human vaccination efforts. This veterinary-surveillance-driven approach has demonstrated the crucial role of animal health monitoring in preventing human outbreaks (Figure 2).

In South Korea, regional pig farms serve as sentinel sites, triggering targeted vector control upon detection of seroconversion in piglets [125]. These findings are reported to the Korea Disease Control and Prevention Agency, which may recommend accelerated human vaccination in nearby communities [125,126].

In Vietnam and Cambodia, pilot projects have connected pig vaccination and mosquito vector monitoring using mobile data collection platforms to strengthen field detection and public health actions [127,128]. These projects exemplify how veterinary interventions can directly contribute to zoonotic disease prevention in humans.

### 5.2. Overcoming Fragmented Responses

Fragmented control efforts risk inefficiency. Effective JE prevention requires integrated management of vaccination, vector control, and animal infections [7,129]. Similarly, mosquito control efforts not informed by real-time animal surveillance data may be misaligned with actual transmission dynamics [40].

Comprehensive control of JE requires integrating veterinary infectious disease management—such as pig surveillance and vaccination—with public health and vector control efforts [130,131]. This convergence of efforts allows for better anticipation of outbreak seasons and more effective resource utilization [40,130]. Geographic information systems and digital surveillance tools can further enhance this integration by mapping risk zones and monitoring intervention coverage in real time [132].

In countries such as China and India, emerging policy initiatives are beginning to link the agricultural and health sectors through joint vector-borne disease control programs [11]. These programs promote information sharing between ministries, training of cross-disciplinary teams, and investment in joint outbreak preparedness systems [11,133,134]. Such cross-sectoral collaborations reflect the practical application of One Health principles in veterinary infectious disease control.

### 5.3. Climate Change and Vector Expansion

**Figure 3:**
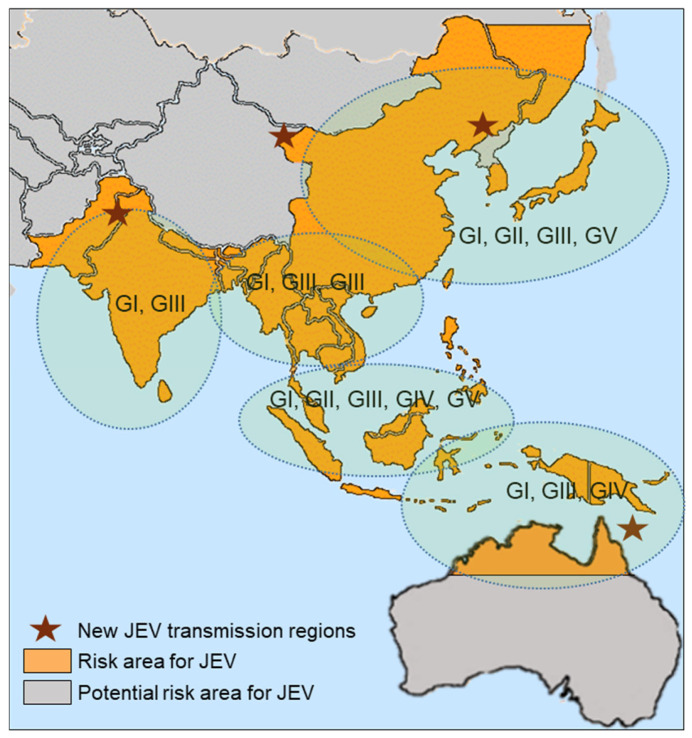
Climate-driven expansion of JEV genotypes I-IV.

Climate change drives shifts in vector distribution and abundance, altering JE transmission patterns [135]. These ecological shifts have resulted in the emergence of JEV transmission in previously non-endemic areas, including parts of northern China, Nepal, and the temperate zones of Australia [136,137] (Figure 3).

In response to these challenges, adaptive strategies must be implemented across all sectors [118]. Environmental monitoring systems that track rainfall, humidity, and land use can provide early warnings of mosquito proliferation [138,139]. These data can be integrated with veterinary surveillance data, particularly regarding pig and bird populations, to trigger preemptive vaccination and vector control measures.

Modeling studies have shown that shifting the timing of vaccination campaigns or adjusting vector control intensity based on climate forecasts can improve the efficiency of public health responses [140]. Furthermore, expanding entomological and veterinary surveillance to high-risk ecological transition zones will be critical for predicting and managing future outbreaks [141]. Ultimately, the growing influence of climate change on vector-borne disease dynamics reinforces the need for inter-sectoral coordination, continuous data sharing, and investment in climate-resilient veterinary infectious disease control programs.

### 5.4. Policy-Relevant, Actionable Recommendations

To translate the One Health approach into effective practice, we recommend that policymakers, veterinarians, and public health officials prioritize animal vaccination programs in regions characterized by high pig density and frequent mosquito activity, as these serve as key amplifying hosts and vectors in JEV transmission. Additionally, integrated surveillance pilot projects should be implemented in areas identified through risk mapping based on climate variables, vector presence, and livestock distribution to enhance early detection and response.

Cross-sectoral collaboration among public health authorities, veterinary services, and environmental agencies is crucial to ensure timely data sharing and coordinated responses to outbreaks. Finally, developing clear guidelines that delineate roles and responsibilities for stakeholders will facilitate efficient resource allocation and effective intervention implementation.

By adopting these targeted strategies, stakeholders can improve early detection, optimize vaccination coverage, and ultimately reduce the risk of JEV transmission more effectively.

## 6. Challenges and Future Directions

### 6.1. Viral Evolution and Vaccine Escape

JEV continues to evolve, with recent studies documenting the emergence of new viral genotypes that differ antigenically from classical strains [15]. This genetic evolution raises concerns about the potential for vaccine escape, in which current vaccines may become less effective against emerging variants [13,25]. For instance, a viral encephalitis outbreak in Australia between 2021 and 2022 was caused by a JEV GIV infection [15], marking the first human outbreak of this genotype since its initial isolation in Indonesia in the late 1970s [16]. As of November 2022, this outbreak has resulted in forty-seven cases and seven deaths [15]. JEV GIV is considered the youngest viral lineage, having emerged approximately 122 years ago, and it evolves rapidly [15]. The emergence of JEV GV in South Korea and China since 2009 also presents challenges, as the current vaccines, predominantly based on JEV GIII, may offer reduced protection against these newer strains [14,142,143]. Specifically, studies suggest that anti-JEV GIII antibodies, produced by available commercial vaccines or non-GV JEV infections, induce less efficient immunity against JEV GV because of their distinct antigenicity [17,143]. Despite the cross-protective immunity demonstrated by the widely used SA14-14-2 live–attenuated vaccine and other formulations against various genotypes, ongoing viral evolution necessitates continuous monitoring [144]. This highlights the need for updated vaccine formulations and booster strategies to ensure sustained effectiveness against emerging variants.

### 6.2. Surveillance Inequity

Effective control of JE is significantly hindered by substantial disparities in surveillance capacity across endemic regions [145]. Many low-resource settings face challenges, including suboptimal laboratory surveillance components, which limit the timely detection of viral activity in animal reservoirs and vectors [146,147]. These gaps contribute to the underreporting of cases and complicate risk assessments in human populations. For example, a 2023 study in China analyzed economic-related inequality in six infectious diseases, including those with transmission routes similar to JE, indicating existing disparities [148]. In low-resource settings, there is a clear need for better data on the burden of encephalitis to improve diagnosis, surveillance, treatment, and prevention [146]. The timely response of phone-based surveillance systems can also be challenged in these environments [149]. To address these issues, efforts to standardize surveillance protocols and expand laboratory capacities are crucial [150]. Mobile diagnostics and community-based surveillance initiatives show promise in bridging these gaps [151]. Portable, real-time, on-site mobile devices and lateral flow assays have been developed for the serodiagnosis of JEV, offering potential solutions for improved surveillance and outbreak prevention in humans [151,152]. Additionally, point-of-care ultrasound has been explored as a diagnostic tool in low-resource settings, although challenges in access and expertise persist [153]. The feasibility of using pig oronasal secretions collected by chewing ropes has also been explored as a non-invasive alternative method for JEV surveillance in epidemic areas, offering viral RNA positivity rates similar to mosquito samples [154]. However, sustainable funding and political commitment remain essential for the successful implementation and scaling of such initiatives [155].

Efforts to standardize surveillance protocols and expand laboratory capacities are crucial for overcoming these challenges. Mobile diagnostics and community-based surveillance initiatives have shown promise in bridging surveillance gaps; however, sustainable funding and political commitment remain essential.

### 6.3. Building Integrated Response Systems

The intricate transmission dynamics of JE necessitate coordinated action across veterinary, entomological, and public health sectors [3]. Despite the widespread recognition of the One Health framework, operationalizing such integrated systems remains challenging because of institutional silos, data sharing limitations, and resource constraints [156]. Developing interoperable surveillance platforms that combine animal health, vector abundance, and human case data can significantly improve early warning and response effectiveness for zoonotic diseases, such as JE [115]. These platforms facilitate coordinated surveillance, which can identify early warning signals for emerging zoonotic diseases [115,157]. The digitalization of surveillance data further provides a foundation for data integration and interoperability across sectors.

Successful examples from East Asia demonstrate that joint task forces, cross-sector training, and shared outbreak preparedness plans significantly enhance control outcomes [134]. The COVID-19 pandemic underscored the importance of the One Health approach in preventing future public health crises, emphasizing the need to consider the health of humans, animals, and the environment [158]. In China, zoonotic disease surveillance involves numerous agencies, highlighting the need for sufficient coordination and interoperability [159]. The adoption of digital solutions and empowered health agents, as seen in Foz do Iguaçu, Brazil, can lead to increased notifications and real-time data for timely decisions, enhancing preparedness for endemic and emerging threats [160]. Scaling these integrated response models globally requires international collaboration, capacity building, and policy support that prioritizes preparedness for zoonotic disease [158,160]. Investing in these integrated response systems not only improves JE control but also strengthens resilience against emerging zoonoses [157,158,159].

## 7. Conclusions

JEV is fundamentally a veterinary infectious disease sustained in animal reservoirs and transmitted to humans through zoonotic pathways. Controlling JEV infection in animal populations remains a fundamental component in preventing human cases. Given that animals such as pigs serve as key amplifying hosts, managing infection among these veterinary reservoirs directly influences the risk of viral transmission to humans.

Surveillance systems that monitor animal infections, particularly in pigs, and vector populations provide critical early warnings that enable timely public health interventions and help to predict outbreaks. For instance, monitoring JEV activity in mosquitoes and swine has been crucial in identifying the spread of the virus to previously non-endemic areas, such as Tibet, highlighting the need for prevention and control strategies alongside surveillance.

Vaccination strategies targeting both humans and animals synergistically reduce the overall disease burden. Human vaccination is considered the most effective and sustainable measure for JE prevention, with different types of vaccines available globally, including inactivated mouse-brain-derived, live–attenuated cell-culture-derived SA 14-14-2, and inactivated cell-culture-derived P3 vaccines. Significant progress has been made in expanding the use of available vaccines and integrating them into routine immunization programs in endemic regions.

However, sustainable JE control requires addressing the infection reservoir in veterinary populations. Controlling viral amplification in pigs and birds not only protects animal health but also breaks the zoonotic transmission cycle to humans. The integration of these approaches within a One Health framework—a collaborative effort across multiple disciplines, including human, animal, and environmental health—strengthens the capacity to anticipate and respond to outbreaks by bridging the gaps between the veterinary, environmental, and human health sectors. This multidisciplinary coordination is essential given the complex eco-epidemiology of JEV and its potential to spread to new ecological niches through climate and ecological changes.

As ecological and climatic changes continue to alter the landscape of vector-borne diseases, prompting the spread of the virus to new populations, it is becoming increasingly important to adopt coordinated, multi-sectoral strategies. Sustained investment in comprehensive surveillance, effective vaccine deployment, particularly new and affordable vaccines for pediatric use, and cross-sector collaboration will be essential to achieve long-term control and minimize the impact of JE on public health. Further research into novel veterinary control methods, such as pig vaccination strategies, ecological vector management, and innovative surveillance tools, will be key to reducing zoonotic transmission risks. Moreover, the development of integrated response platforms that link veterinary and human disease management will strengthen preparedness against emerging zoonotic threats.

## Figures and Tables

**Table 1:** Comparison of animal and human surveillance systems for JE by country.

Country	Main Animal Surveillance Targets	Animal Surveillance Methods	Human Surveillance Methods	Responsible Agencies	Surveillance Frequency and Coverage	References
Japan	Pigs and mosquitoes	Serological testing in pigs, mosquito trapping, and virus isolation	National notifiable disease reporting and clinical surveillance	Ministry of Agriculture, Forestry and Fisheries; Ministry of Health	Year-round continuous monitoring focused on key regions	[44,45]
South Korea	Pigs and wild birds	Pig antibody monitoring and PCR testing in wild birds	Mandatory disease reporting and clinical diagnosis	Korea Disease Control and Prevention Agency; Animal and Plant Quarantine Agency	Seasonal focused surveillance prioritizing high-risk areas	[46,47]
China	Pigs, mosquitoes, and birds	Pig serology, mosquito virus isolation, and bird antibody testing	Hospital-based surveillance and local health reporting	National Health Commission; Ministry of Agriculture	Seasonal monitoring with regional variation	[48,49]
Vietnam	Pigs and mosquitoes	Pig antibody testing and mosquito virus surveillance	Local health center reporting and clinical surveillance	Ministry of Health; Ministry of Agriculture and Rural Development	Seasonal surveillance focused on high-risk zones	[50,51,52]

**Table 2:** Comparison of human and animal JE vaccines and vaccination strategies.

Vaccine Type	Target Population	Administration Route	Dosage and Schedule	Duration of Immunity	Cost-effectiveness and Characteristics	References
SA14-14-2 (Live–Attenuated)	Humans and pigs	Subcutaneous or intramuscular injection	Humans: 1–2 doses; pigs: seasonal vaccination recommended	Humans: over 5 years; pigs: 4–6 months	Low cost; strong immune response; standard vaccine in some countries	[1,85,86,87]
Vero Cell-Derived Inactivated Vaccine	Humans	Intramuscular injection	3-dose primary series with boosters recommended	Approximately 3–5 years	High safety profile; recommended for travelers and high-risk groups	[88,89,90]
JE-CV (Live Chimeric Vaccine)	Humans	Subcutaneous injection	Single dose with long-lasting immunity	Over 5 years	Rapid immune response; fewer side effects; increasingly adopted	[91,92,93,94]
Inactivated Animal Vaccine	Pigs	Intramuscular injection	Routine vaccination before production or reproduction	4–6 months	Aimed at improving productivity; limited use in some regions	[95,96]
